# Gatekeepers or barriers? Exploring traditional medicine practitioners’ understanding of breast cancer in Ghana

**DOI:** 10.1371/journal.pone.0355289

**Published:** 2026-08-05

**Authors:** Perpetual Arthur, Adwoa Bemah Boamah Mensah, Kofi Boamah Mensah, Thomas Okpoti Konney, John Amuasi, Shalini Kulasingam, Beth A. Virnig

**Affiliations:** 1 Department of Nursing, School of Nursing and Midwifery, Kwame Nkrumah University of Science and Technology, Kumasi, Ghana; 2 Kumasi South Regional Hospital, Ghana Health Service, Kumasi, Ashanti Region, Ghana; 3 Department of Pharmacy Practice, Faculty of Pharmacy and Pharmaceutical Sciences, Kwame Nkrumah University of Science and Technology, Kumasi, Ghana; 4 Department of Obstetrics and Gynaecology, School of Medical Sciences, Kwame Nkrumah University of Science and Technology, Kumasi, Ghana; 5 Global One Health Research Group, Bernhard Nocht Institute of Tropical Medicine, Hamburg, Germany; 6 Department of Global Health, School of Public Health. Kwame Nkrumah University of Science and Technology, Kumasi, Ghana; 7 Global Health and Infectious Diseases Research Group, Kumasi Centre for Collaborative Research in Tropical Medicine, UPO PMB, KNUST, Kumasi, Ghana; 8 Celia Scott Weatherhead School of Public Health and Tropical Medicine, Tulane University, New Orleans, Louisiana, United States of America; 9 College of Public Health and Health Professions, University of Florida, Gainesville, United States of America; TIU: Tishk International University, IRAQ

## Abstract

**Background:**

In Ghana, traditional medicine practitioners (TMPs) are often the first point of contact for women with breast health concerns. However, they operate independently of formal healthcare services. While their embeddedness within communities grants them cultural legitimacy, their perceptions of breast cancer remain underexplored. Understanding TMPs’ explanatory models, recognition of signs, and diagnostic practices is crucial for improving early detection of breast cancer and reducing delays in accessing treatment that lead to poor outcomes.

**Methods:**

We conducted a qualitative exploratory descriptive study using semi-structured interviews with 14 non-formally trained TMPs involved in breast care services in Ghana. Interviews were transcribed, translated, and thematically analyzed to understand how they recognize and interpret symptoms, their beliefs about the causes and risks of breast cancer, and how they diagnose and treat breast-related problems.

**Results:**

Our analysis generated four major themes: sources of knowledge, multi-dimensional explanations of breast cancer causation, cardinal signs defining breast cancer recognition, and experiential and practice-based approaches to diagnosis. TMPs relied on inherited ancestral traditions but also sought to improve their skills through workshops, professional associations, and occasional biomedical collaborations, showing a willingness to learn and adapt to new information. However, significant misconceptions remained, including beliefs that spiritual or social disharmony and reproductive and lifestyle factors cause breast cancer. While TMPs identified key signs such as lumps, nipple changes, and skin alterations, their dependence on experiential and intuitive diagnostic practices risked both over- and under-diagnosis.

**Conclusion:**

TMPs are central actors in Ghana’s breast cancer pathway, representing both opportunities for early recognition and risks of misinformation. Correcting misconceptions and embedding TMPs into structured training and referral systems could reduce diagnostic delays, enhance early detection, and support Ghana’s National Cancer Control Plan and Sustainable Development Goal 3.

## Introduction

Breast cancer (BC) is a significant global public health concern, with an increasing incidence and disproportionate mortality in low- and middle-income countries (LMICs), including Ghana [1]. While the incidence rates are comparatively lower in LMICs than in high-income countries, mortality rates are markedly higher due to a combination of factors, including delayed diagnosis, limited access to specialized care, and the reliance on non-biomedical health systems [2,3].

In Ghana, BC is the most common cancer among women and a leading cause of cancer-related deaths [4]. A majority of cases (80%) are diagnosed at advanced stages, often after a prolonged time from symptom onset to formal diagnosis [4,5]. The delays are partly attributed to the absence of a national screening program coupled with structural weaknesses in the formal healthcare system, but they are also profoundly influenced by sociocultural norms and health-seeking behaviors that prioritize traditional medicine as a first-line response [3,4].

Several studies have reported that traditional medicine, covering herbal remedies and faith or spiritual healing, deeply rooted in Ghanaian society, plays a vital role in the health journeys of women with breast-related symptoms. Boamah Mensah et al. [3] reported that women often turn to traditional medicine practitioners (TMPs) driven by considerations of cost-effectiveness, fear of invasive procedures, spiritual explanations of illness, and delays in the orthodox healthcare system. Mburu et al. [5] demonstrated that women follow nonlinear pathways to care, beginning with self-detection and then consulting TMPs before seeking hospital care. Bonsu et al. [4] documented that women frequently misjudge early symptoms (such as painless lumps or bloody nipple discharge) and delay care until symptoms worsen, often influenced by social networks and TMPs who either downplay the severity or reframe the symptoms in spiritual or benign terms.

Despite their widespread influence, non-formally trained TMPs, including herbalists and faith healers, are largely excluded from Ghana’s formal cancer detection and referral systems. They operate independently of the Orthodox healthcare services. Existing efforts by the Ministry of Health to institutionalize traditional medicine focus on formally trained practitioners, leaving the majority of non-formally trained TMPs, who are often the first point of contact for women with breast concerns, outside the scope of cancer control policies and collaborations [6,7]. This disconnect is a missed opportunity to engage a key group of frontline providers who could potentially play a pivotal role in promoting early breast cancer detection, guiding appropriate referrals, and dispelling misinformation about BC while providing spiritual support.

Few studies have explored how non-formally trained TMPs conceptualize breast cancer. Their beliefs about its causes, signs and symptoms, and diagnostic approaches remain largely undocumented. This knowledge gap is particularly concerning because these practitioners often serve as the initial interpreters of symptoms and may offer herbal or spiritual treatments rather than encourage biomedical care. Understanding the diagnostic logic and treatment pathways used by TMPs is therefore crucial for designing culturally grounded interventions that promote earlier diagnosis and enhance cross-system collaboration.

This study addresses this gap by exploring the perspectives of non-formally trained TMPs around breast cancer in Kumasi, Ghana. Specifically, it investigates how they recognize and interpret symptoms, their beliefs about the causes and risks of breast cancer, and how they diagnose and treat breast-related problems. Amplifying the voices of TMPs provides important insights to develop training programs, referral systems, and strategies for integrating traditional and biomedical approaches. Ultimately, this can help reduce diagnostic delays and improve breast cancer outcomes in Ghana.

## Materials and Methods

### Study design and setting

This study examined the perspectives of traditional medicine practitioners (TMPs) and faith healers on breast cancer, focusing on its causes, main signs and symptoms, and methods of diagnosis. An exploratory, descriptive, qualitative design was employed, utilizing in-depth, semi-structured interviews to gather participants’ experiences [8]. The study was carried out in Kumasi, Ghana, with participants recruited from communities within the Kumasi Metropolitan Assembly (KMA).

### Study population and eligibility

We targeted TMPs involved in breast health and BC care in Kumasi, Ghana. In this context, traditional medicine includes the use of medicinal plants and faith-based or spiritual healing [9,10]. This study defines TMPs as non-formally trained herbalists or faith healers recognized within their community for providing care based on traditional knowledge, practices, and beliefs. We included men and women aged 18 years or older who were involved in women’s breast health practices within the Kumasi Metropolitan Assembly (KMA), had at least 1 year of experience in breast health or cancer care, could speak either Twi or English, and were willing to participate. Formally trained TMPs were excluded from the study.

### Sampling and sample size

We used purposive sampling to recruit participants who were willing to participate and able to share their experiences [8]. Fourteen TMPs were interviewed, with the sample size (n = 14) determined by data saturation. During concurrent data collection and analysis, later interviews did not add substantively new insights relevant to the research questions. By the 12th participant, responses were repetitive, and two additional interviews were conducted to confirm saturation [11–13].

### Recruitment and the interview process

Before commencing the study, formal permission was obtained from the study site via an introductory letter and an ethics approval letter. Participants were recruited from communities within the Kumasi Metropolis via the Traditional Medicine Practice Council (TMPC). Two TMPC staff members served as “recruitment links”, providing potential participants with study information, eligibility criteria, and information leaflets. Interested TMPs were then referred to the first author (P.A.), who confirmed eligibility, discussed study details, and scheduled interview appointments. Data collection took place from 10^th^ February to 30th April 2023 through in-depth, face-to-face interviews guided by a semi-structured interview guide [14]. The guide (S1 File), developed based on the study objectives and existing literature, was reviewed by two TMPs with expertise in breast health and then translated into Twi using a back-to-back translation process. It was piloted with two herbal/faith healers to refine the questions; these pilot interviews were excluded from the primary analysis. Interviews were conducted by the first author (P.A.), a nurse trained in qualitative research, in Twi at participants’ chosen locations, usually their own centers, for privacy and comfort. Sessions lasted 35–60 minutes (averaging 45) and were audio-recorded with participants’ consent. Field notes capturing nonverbal cues and the researcher’s reflections were also taken. Written consent was obtained before participation. Demographic information from participants was also collected. Participants’ anonymity was maintained by assigning unique study codes (e.g., TMP001–TMP014), and they were reminded of their right to withdraw at any time.

### Data analysis

Data collection and analysis were conducted concurrently. Interviews were transcribed from Twi into English by a professional transcriber proficient in both languages. A bilingual author (A.B.B.M.) cross-checked a random sample of the recordings and transcripts to support accuracy in interviewing, transcription, and translation. Transcripts were imported into NVivo 12.0 Plus [15] for data management and analysis. We used an inductive thematic analysis approach, developing codes and candidate themes through iterative engagement with the transcripts rather than applying a pre-existing theoretical framework [16,17]. Two analysts (A.B.B.M. and P.A.) independently read the transcripts, generated initial codes, and discussed analytic interpretations to refine broader categories and themes. Differences in interpretation were discussed with a senior author (B.V.) to deepen the analysis and enhance transparency. Themes were refined through constant comparison across transcripts, team debriefings, and repeated cross-checking with the data to strengthen credibility and confirmability [18,19]. By the 12th interview, additional interviews were not adding substantively new insights relevant to the research questions; two additional interviews were conducted to assess the dataset’s adequacy and depth [12]. Final themes were integrated and supported with illustrative participant quotes to preserve authenticity and transferability [20,21].

### Trustworthiness

Credibility was supported through analyst triangulation and member checking. Analyst triangulation involved two authors (A.B.B.M. and P.A.) independently coding transcripts and comparing interpretations to refine the analytic account [22]. To enhance confirmability, the interviewer summarized each session for participants immediately after the interviews, and three randomly selected participants later reviewed their transcripts, all confirming accuracy without corrections [23]. Field notes documenting nonverbal cues, participants’ concerns, and the researcher’s reflections further strengthened dependability. Although the interviewer (P.A.) is a graduate nurse with clinical knowledge of breast cancer, she had no professional ties to the study setting or participants, which helped reduce potential bias.

### Ethical approval and consent to participate

This study was approved by the Ghana Health Service Ethical Review Board with approval number [GHS-ERC: 007/09/22]. The participants provided written consent. All study methods were carried out in accordance with the ethical principles outlined in the 2013 World Medical Association Declaration of Helsinki [24]. All participants were informed about the study’s purpose, assured of confidentiality, and provided written consent before participating. Participation was voluntary, and participants could withdraw at any time without consequence. Participants were assured of anonymity using study numbers (codes) throughout the study and during the verbatim quotations of participants’ expressions. We de-identified transcripts before analysis to ensure anonymity. Audio recordings and transcripts (without any identifying information) were stored on a password-protected computer. We followed the Standards for Reporting Qualitative Research [25].

## Results

### Participant’s profile

Fourteen herbal/faith-based healers participated in the study. Six were solely herbalists, while eight practiced both as herbalists and as spiritual (faith-based) healers. Table 1 presents detailed demographic characteristics, providing important context for understanding the diversity of backgrounds, experiences, and therapeutic approaches that shape their perspectives on breast cancer care within the Ghanaian traditional healing setting.

**Table 1:** Participants’ Background Data.

Participant ID	Age range (years)	Sex	Religion	Marital status	Level of education	Years in profession	Mode of training	Unit/department
TMP 001	40-50	Male	Muslim	Married	JHS	20	Apprenticeship	Herbal medicine
TMP 002	51-60	Male	Christian	Married	Middle school	30	Apprenticeship	Herbal/Spiritual
TMP 003	40-50	Male	Christian	Single	Tertiary	6	Apprenticeship	Herbal/Spiritual
TMP 004	30-39	Female	Christian	Married	Tertiary	5	Apprenticeship	Herbal/ Spiritual
TMP005	40-50	Male	Christian	Married	No formal education	3	Apprenticeship	Herbal medicine
TMP006	30-39	Male	Christian	Married	SHS	15	Apprenticeship	Herbal/Spiritual
TMP007	40-50	Male	Christian	Divorced	SHS	32	Apprenticeship	Herbal/Spiritual
TMP008	61-70	Male	Christian	Married	Middle school	32	Apprenticeship	Herbal medicine
TMP009	40-50	Male	Christian	Married	SHS	10	Apprenticeship	Herbal medicine
TMP010	30-39	Male	Christian	Divorced	SHS	17	Apprenticeship	Herbal/Spiritual
TMP011	40-50	Male	Muslim	Married	Tertiary	20	Apprenticeship	Herbal medicine
TMP012	40-50	Male	Christian	Married	SHS	15	Divine (from the holy spirit)	Herbal/Spiritual
TMP013	40-50	Male	Christian	Married	Tertiary	8	Apprenticeship	Herbal medicine
TMP014	330−39	Male	Traditionalist	single	Tertiary	13	Apprenticeship	Herbal/ Spiritual

### Thematic findings

Thematic analysis of the interviews identified four main themes: (i) sources of knowledge, (ii) multi-dimensional explanations of breast cancer causation, (iii) cardinal signs defining breast cancer recognition, and (iv) experiential and practice-based approaches to diagnosis. These themes and their sub-themes as shown in Table 2, offer insights into how TMPs understand breast cancer and the practices they use when responding to women’s breast-related concerns.

**Table 2:** Themes and Sub-themes.

Themes	Sub-themes
Sources of knowledge	i. Inherited and ancestral knowledge ii. Blending traditional knowledge with formal and professional training
Multi-dimensional explanations of breast cancer causation	i. Spiritual and social disharmony ii. Bodily vulnerability and lifestyle practices.
Cardinal signs defining breast cancer recognition	i. Abnormal lumps and swelling as the primary indicators. ii. Nipple and skin changes as confirming signs.
Experiential and practice-based approaches to diagnosis	i. Hands-on experiential assessment ii. Reliance on external biomedical confirmation.

## Theme #1: Sources of knowledge

TMPs’ understanding of BC is shaped by diverse knowledge pathways. Some rely on inherited and ancestral wisdom, passed down through family lineage and cultural traditions. Others blend traditional knowledge with formal training and professional engagement. Together, these pathways illustrate how TMPs construct authority and credibility in their breast cancer care practice. Two subthemes emerged: inherited and ancestral knowledge and blending traditional knowledge with formal and professional training.

### Inherited and ancestral knowledge

TMPs’ understanding of breast cancer is rooted in knowledge passed down through generations, often from grandparents, parents, or family elders who practiced herbalism, spiritual healing, or both. This lineage-based transmission gives legitimacy and authority to their practices, combining cultural, spiritual, and experiential learning rather than formal biomedical training:

*“I received the medicine from my grandmother; it was a gift. She healed cancer.” (TMP004)*

*“I learned it from my grandfather, … My grandfather was a pastor, and my father was a Mallam and a fetish priest. So, they all have their own ways of preparing their medicine.” (TMP006)*

*I don’t have any spirit. I only followed in my grandfather’s footsteps, and he taught me about the medicinal value of various natural herbs. (TMP014)*

### Blending traditional knowledge with formal and professional training

Some TMPs enhance their inherited or community-based knowledge through training, workshops, professional associations, and collaborations with biomedical practitioners. This evolving model is combines traditional healing with elements of modern healthcare, reinforcing legitimacy and expanding their skills:

*“..., we do go through training courses. Occasionally, we self-fund an event for a nurse to come and teach us something. In addition to that, I have four doctors that I consult...” (TMP012)*

*“Every year, we attend training sessions. We are part of an organization called the Ghana National Traditional Medicine Practice Association.” (TMP001)*

*“I started with a family member, then I went to school and began reading books and other things. ... Yes, I attended a workshop on herbal medicine.” (TMP003)*

## Theme #2: Multi-dimensional explanations of breast cancer causation

TMPs interpret BC through a multi-layered explanatory framework that combines spiritual, social, and physical understandings of disease. Illness is often viewed as stemming from spiritual or moral disruptions, such as curses, dreams, or broken social obligations, or from bodily vulnerabilities related to reproductive health, infections, hygiene, or lifestyle practices. This holistic worldview sees BC as both a spiritual and physical condition, influencing how TMPs advise clients, choose treatments, and decide when referral to biomedical care is needed. Two sub-themes emerged: (i) spiritual and social disharmony and (ii) bodily vulnerability and lifestyle practices.

### Spiritual and social disharmony

TMPs viewed BC as a problem caused by spiritual imbalance or disrupted social connections. They believed that dreams, curses, and unresolved conflicts often precede physical symptoms, suggesting that the illness is more than a medical condition—it is a moral or spiritual issue that needs reconciliation or ritual healing:

*“Many people who come here say they dreamt that someone bit their breast… and before they wake, they start to notice a change in their breast. Most sickness begins spiritually before manifesting physically.” (TMP013)*

*“A woman came here with a breast problem. I told her it was not just a disease, breast cancer; she wronged someone, and she must apologize to be healed.” (TMP008)*

### Bodily vulnerability and lifestyle practices

Some TMPs attributed BC to physical vulnerabilities linked to reproductive health, infections, poor hygiene, or lifestyle choices. Menstrual irregularities, abortions, sexually transmitted diseases, smoking, dietary habits, and even clothing practices were seen as weakening the body and creating conditions for disease, reflecting a holistic but non-biomedical understanding of risk:

*“When they have menstrual problems or undergo late abortions, it causes many issues in the breast... those with menstrual problems may experience some gas in the breast, which can lead to pain, then cancer.” (TMP001).*

*“Nine out of ten breast cancer patients who visit my clinic have infectious diseases like gonorrhea, hepatitis B, syphilis…” (TMP014).*

*“If someone is complaining about her breasts, we usually examine her cervix because they are linked… If there’s a sore on the cervix, it can affect the breast, so I make sure to treat any infections in the cervix first before addressing the breast.” (TMP005)*

*“…, things like smoking and nipple biting during sex cause cancer.” (TMP011)*

*“Someone will use one brassiere for a long time... the blood does not circulate well, and they get breast cancer.” (TMP004)*

*“Eating foods high in fats can harm your health and make you more likely to get sick often. Too much of anything is unhealthy. Taking in too much fat is unhealthy.” (TMP004)*

## Theme #3: Cardinal signs defining breast cancer recognition

TMPs identify BC mainly through observable and tangible signs they see as diagnostic of the disease. Key are abnormal lumps and swelling in the breast, along with changes in the nipple and skin. For TMPs, the presence of these cardinal signs provides enough evidence to conclude that a woman has breast cancer, reflecting a symptom-focused model of diagnosis that relies on visible and physical signs of illness. Two sub-themes were identified: (i) abnormal lumps and swelling as the primary indicators and (ii) nipple and skin changes as confirming signs.

### Abnormal lumps and swelling as primary indicators

For many TMPs, the presence of an abnormal lump or swelling are perceived as conclusive markers of disease.

*“The breast will resemble the oval mouth of a deodorant, or other things, like an orange... For those with a lump in their breast, it becomes swollen... clearly indicating that the person has breast cancer.” (TMP013)*

*“Any lump other than the natural one is abnormal” (TMP001).*

*“We know it is painless. There is no pain or lump in the breast. So, based on the presenting symptoms, we can conclude it is cancer” (TMP012).*

### Nipple and skin changes as confirming signs

Beyond lumps, TMPs identified visible changes in the nipple including nipple retraction, hardness, loss, abnormal discharge, skin discoloration, rashes, sores, and peeling textures as clear evidence that the breast was diseased.

*“You will notice that the nipple is either missing, diverted, or feels very hard” (TMP007).*

*“Some also notice that the area around the nipple changes color. Some may not be breastfeeding, but there is discharge from the nipple; all these signs indicate breast cancer.” (TMP012).*

*“Sometimes, you will see rashes and soreness around the breast, and at times, the texture of the breast feels like it is peeling away.” (TMP014).*

## Theme #4: Experiential and practice-based approaches to diagnosis

For TMPs, breast cancer suspicion was grounded in direct physical assessment, such as palpating lumps or observing visible bodily changes, and given credibility by years of practice. Some practitioners also relied on biomedical confirmation through hospital scans or patient reports. Although the interviews did not systematically trace patients’ full care pathways after recognition or suspicion, some accounts suggested that TMPs’ beliefs about causation shaped their immediate management responses. Two sub-themes were identified: (i) hands-on experiential assessment and (ii) reliance on external biomedical confirmation.

### Hands-on experiential assessment

TMPs stressed that their ability to diagnose relies on accumulated experience and direct physical examination of the breast. Using the hands to feel for lumps or to observe visible changes was considered sufficient to suspect BC. This reliance on practical knowledge positioned “experience as the best teacher,” with credibility based on years of practice rather than formal education.

“*We assess the breast and any lump; aside from the natural lumps are considered abnormal. If you have much experience in breast cancer, you will know. For me, I use my hand to check it.” (TMP001)*

*“I will check their body and physical appearance. You will see they [breasts] have become smaller than they should be, so if we notice that, we check with our hands to confirm if it is cancer” (TMP005).*

*“For me, I oppose using gloves to examine the breast, so I usually use my hand to check for lumps. It might even surprise you that the situation isn’t even cancer in the end.” (TMP006)*

*“...because I have a lot of experience with this, when they visit, I check their breast to determine it.” (TMP001)*

*“Experience is the best teacher, and I have been doing this business for a long time” (TMP014).*

### Reliance on external biomedical confirmation

Although many TMPs relied on experiential methods, some acknowledged the role of biomedical tests and hospital diagnoses in confirming breast cancer. In such cases, practitioners either deferred to hospital scans or relied on patients bringing medical reports, which they used to validate their own suspicions. This blending of experiential knowledge with biomedical confirmation illustrates pragmatic hybridity in diagnostic reasoning.

“We only suspect breast cancer when we see that, but the scan will confirm it.” (TMP004)

*“I typically see patients who have been to the hospital and whose doctors have already confirmed she has breast cancer. Such a person will come with her cancer results in hand.” (TMP014)*

### Analytical paragraph: Management responses after recognition or suspicion

Although the study did not systematically trace patients’ full care pathways after TMP recognition or suspicion of breast cancer, some accounts suggested that practitioners’ beliefs about aetiology shaped their immediate management responses. Where breast symptoms were interpreted through spiritual or social explanations, management could involve advice directed at spiritual or relational repair. For example, one practitioner explained: *“A woman came here with a breast problem. I told her it was not just a disease, breast cancer; she wronged someone, and she must apologize to be healed” (TMP008).*

Similarly, where breast cancer was attributed to bodily vulnerability, reproductive problems, or infection, management could focus first on treating those perceived underlying causes. One TMP stated: *“If there’s a sore on the cervix, it can affect the breast, so I make sure to treat any infections in the cervix first before addressing the breast” (TMP005).* These accounts suggest that aetiological beliefs informed how some TMPs advised or managed women with breast symptoms. However, other TMPs described a more cautious approach, recognizing suspicious signs but relying on biomedical confirmation*: “We only suspect breast cancer when we see that, but the scan will confirm it” (TMP004).* Another practitioner explained that some women came after hospital confirmation: *“I typically see patients who have been to the hospital and whose doctors have already confirmed they have breast cancer. Such a person will come with her cancer results in hand” (TMP014).*

Together, these findings indicate that TMP responses after recognition ranged from traditional or spiritually informed management to selective reliance on biomedical confirmation.

## Discussions

This study provides new insights into how non-formally trained traditional medicine practitioners (TMPs) in Kumasi, Ghana, conceptualize breast cancer. TMPs are deeply embedded within communities and are often the first point of contact for women with breast concerns, a role consistent with evidence from across Africa showing that traditional healers are central to health-seeking pathways [22,23]. Their influence makes them a critical resource and a potential barrier for early detection and timely diagnosis.

A major finding of this study is the diversity of knowledge pathways through which TMPs construct their authority. Some drew legitimacy from inherited and ancestral wisdom, passed down through family lines, while others actively sought to expand their knowledge by joining professional associations, attending workshops, and even inviting biomedical practitioners to deliver training on breast cancer. This intentional pursuit of knowledge reflects not resistance but openness to biomedical engagement. Similar patterns have been observed in other contexts, where healers strive to integrate traditional and modern expertise to refine their skills [22,23]. This finding suggests that TMPs could be valuable partners in community-based cancer education and referral if their readiness to learn is systematically supported. Harnessing this willingness through structured training could create sustainable bridges between traditional and biomedical systems.

Nonetheless, the study also uncovered significant misconceptions in TMPs’ explanatory models of breast cancer causes. Some practitioners believed the disease was due to spiritual or social disharmony, while others pointed to reproductive and lifestyle factors such as menstrual irregularities, abortions, nipple biting, or sexually transmitted infections. These beliefs are not supported by biomedical evidence and demonstrate a limited understanding of known risk factors. Similar misconceptions have been recorded elsewhere in Ghana and across Africa, where breast cancer is often viewed through spiritual or mystical lenses [3,4,22]. These beliefs may shape how TMPs advise and manage women after recognizing breast symptoms. In our findings, spiritual and social explanations were associated with advice on reconciliation or spiritual resolution, whereas bodily explanations were associated with treatment of perceived reproductive or infectious causes. Such responses could plausibly contribute to delays in biomedical assessment, particularly when breast symptoms are managed first within traditional or spiritual frameworks. However, our study did not directly examine patient pathways after TMP consultation, referral decisions, or time to biomedical diagnosis; therefore, we interpret this as a potential mechanism rather than direct evidence of delay. These results suggest the urgent need for tailored education to correct misinformation and to strengthen TMPs’ role as facilitators rather than barriers to timely care. Such training could guide TMPs in following the health services’ preventive, diagnostic, and care practices. When it came to recognition, TMPs identified what they considered the cardinal signs of breast cancer, including abnormal lumps (painful or painless), swelling, nipple retraction, discharge, and skin or texture changes. These observations partially overlap with biomedical indicators, suggesting that TMPs can recognize suspicious symptoms. However, the conflation of any “unnatural” lump with cancer or the assumption that painful symptoms are diagnostic reveals the risks of both over-diagnosis and under-diagnosis. This reliance on symptom-centered, experiential reasoning aligns with findings from other African contexts, where TMPs use tangible manifestations of disease as diagnostic anchors [23,24]. While such practices strengthen their credibility within communities, they underscore the limitations of experiential authority without biomedical confirmation. Encouragingly, some TMPs acknowledged hospital scans or patients’ medical reports as confirmatory evidence, indicating a pragmatic openness to biomedical authority that could be leveraged in collaborative diagnostic pathways.

These findings align with the broader literature, which demonstrates that TMPs play multiple roles across cancer care, from diagnosis and treatment to psychosocial support and palliation [22]. On the one hand, their practices may delay biomedical care, as seen in studies from Ghana and Nigeria, where healer consultations are associated with advanced-stage presentations [24,25]. On the other hand, their community trust and openness to biomedical collaboration make them potential allies. One explanation for this hybridity is that TMPs strategically adopt biomedical language to bolster credibility with clients while maintaining spiritual frameworks to preserve cultural resonance. Alternatively, they may be genuinely integrating traditional and biomedical logics into a syncretic worldview. Either way, the outcome highlights both risk and opportunity.

Policy implications are therefore clear. Ghana’s National Cancer Control Plan prioritizes early detection, timely diagnosis, prompt treatment, and community engagement as key strategies for reducing cancer deaths [26]. Recognizing the important role of TMPs in women’s care pathways, involving them in awareness initiatives and referral systems could help reduce delays. Developing training programs in partnership with TMP associations and biomedical experts can help address misconceptions and provide TMPs with evidence-based knowledge about risk factors and the cardinal signs of the disease. These strategies would respect the cultural authority of TMPs while ensuring patients are timely referred to biomedical care when necessary.

## Conclusions

In conclusion, this study highlights the essential yet complex role of TMPs in Ghana’s breast cancer landscape. Their reliance on traditional knowledge, experiential practices, and spiritual beliefs reflects both cultural continuity and notable gaps in their understanding of biomedical concepts. Misconceptions about causes may delay care, but TMPs’ willingness to receive training and collaborate offers a promising path for integration. By providing TMPs with accurate information and incorporating them into health services’ preventive, diagnostic, and care practices, Ghana can turn them from potential barriers into valuable partners. Such a strategy not only supports the objectives of the National Cancer Control Plan but also aligns with Sustainable Development Goal 3 (Good Health and Well-Being) [27] by reducing premature deaths from non-communicable diseases through inclusive and culturally sensitive approaches. Utilizing TMPs’ influence as trusted community figures could be transformative, improving early breast cancer detection in Ghana and strengthening cancer control efforts across Africa.

### Strengths and limitations

This study has important strengths. It is among the first to systematically explore the perceptions of non-formally trained traditional medicine practitioners (TMPs) in Ghana regarding breast cancer, offering novel insights into their explanatory models, diagnostic practices, and openness to biomedical collaboration. The exploratory descriptive qualitative design, combined with in-depth interviews, enabled the collection of rich, nuanced accounts that quantitative approaches would not have captured. Rigor was ensured through analyst triangulation, member checking, and detailed field notes, which enhanced the credibility and trustworthiness of the findings. Nonetheless, several limitations should be acknowledged. The study was conducted in Kumasi alone and included a relatively small sample of 14 TMPs, which may limit the transferability of the findings to other contexts in Ghana. The reliance on self-reported perceptions also introduces the possibility of social desirability bias, particularly in responses regarding collaboration with biomedical practitioners. At the same time, the translation of interviews from Twi into English may have resulted in a subtle loss of meaning. Further, the study did not include the perspectives of women with breast cancer or biomedical providers, limiting a full understanding of how TMPs’ beliefs influence health-seeking behavior and patient outcomes. In addition, although the study explored TMPs’ recognition, beliefs about causation, and diagnostic practices, it did not systematically examine what happened after TMPs suspected breast cancer. We therefore could not fully assess whether practitioners referred women to biomedical facilities, treated them within traditional systems, advised spiritual or social remedies, or whether these responses contributed to diagnostic delays. Future research should follow patient pathways after TMP consultation and include the perspectives of women, TMPs, and biomedical providers to better understand how aetiological beliefs influence management, referral, and time to diagnosis.

## Supporting information

S1 File Interview guide. (PDF)

## Abbreviations

BC: breast cancer
CHRPE: committee on human research, publication, and ethics
LMICs: low- and middle-income countries
TMPs: traditional medicine practitioners
TMPC: traditional medicine practice council.
