# Supplementary material for: Gatekeepers or barriers? Exploring traditional medicine practitioners’ understanding of breast cancer in Ghana
Source: PLoS One. 2026 Aug 5;21(8):e0355289. doi: 10.1371/journal.pone.0355289 (PMC13440859; doi:10.1371/journal.pone.0355289)
Supplement: Supplementary file 1 [file pone.0355289.s001.pdf]

KWAME NKRUMAH UNIVERSITY OF SCIENCE & TECHNOLOGY

UNIVERSITY OF MINNESOTA
Driven to Discover®

ENGLISH VERSION

Interview Guide

Thank you for agreeing to participate in this interview. I am here to learn from your experiences as a traditional medicine provider/faith healer who treats patients with breast-related health concerns. Your insights are valuable and will make a significant contribution to our research. This interview will take approximately 60 minutes and will be audio-recorded and transcribed**.** Everything you share will remain strictly confidential and used solely for academic purposes. Your identity will not be disclosed at any point. Participation is voluntary, and you may:

i. Refuse to answer any question you're uncomfortable with.
ii. Stop the recording or the interview at any time.

There are no right or wrong answers. We are interested in your perspective and experience. You can stop the interview at any stage. Shall we begin?

Study Interview Guide

Version: July 2022

Section I: Interview Details

1.1 Participant Code Number: | | | | 1.2 Date of interview: |__|__/0| x/2022/3

1.3 Name of Interviewer: ____________________

1.4 Facility Name: __________________________

1.5. Interview Start Time: ______________________

1.6. Interview End Time:

1.7. Name of Data Entry Personnel (DEP):____

ELIGIBILITY CHECK: RESPONDENT MUST BE 18 YEARS OR OLDER

☐ Eligible ☐ Not Eligible → *If not eligible, terminate interview.*

Note: Use code **99** for any question the respondent refuses to answer.

Section II: General demographic information

This first set of questions is general information questions about you.

1. How old are you? (In completed years) …………………………………………………
2. How long have you been practicing as a traditional healer? (years) ………………………
3. What is your religious affiliation?

a. Christianity [ ]
b. Islam [ ]
c. Traditional African religion [ ]

4. What is your highest educational qualification?
   a. No formal education [ ]
   b. Primary education [ ]
   c. Secondary education [ ]
   d. Tertiary education [ ]
5. Do you hold any qualification(s) aside from herbal treatment?
   a. Yes [ ]
   b. No [ ]

   If Yes, please specify: ………………………………………………………
6. What type of traditional services do you offer?
   a. Herbal medicine [ ]
   b. Spiritual (e.g., incantations, sacrifices) [ ]
   c. Other (specify)……………………………………..
7. Do you have any training in herbal/spiritual medicine?
   a. Yes [ ]
   b. No [ ]
8. If Yes, what type of training do you have?
   a. From family members (e.g., father, grandfather, etc) [ ]
   b. Through dreams/visions [ ]
   c. Formal training [ ]
   d. Other (specify)………………………………………………
9. Have you ever attended formal training or workshops on herbal medicine/treatment?
   a. Yes [ ]
   b. No [ ]

11 Do you specialize in any particular area(s) of herbal medicine?
   a. Yes [ ]
   b. No [ ]

   If yes, please specify: …………………………………………………………………

10. On average, how many women do you attend to per month? ………………………………
11. How many new patients do you see monthly? ………………………………
12. Do you maintain patient records?
    a. Yes [ ]
    b. No [ ]
13. What is your usual fee for treatment/medication? ………………………………………
14. Do you accept any form of exchange aside from cash?

a. Yes [ ]
b. No [ ]

If yes, what do you accept? ………………………………………………………………

15. Do/Have you ever referred patients with suspected cancer to hospitals?
    a. Yes [ ]
    b. No [ ]

    If yes, how many referrals per month on average? ……………………………………………

The Interview Questions

Main Question

1. When a woman visits you with a breast complaint, how do you determine the nature of the problem?

Probes:

a. How do you differentiate between cancer and non-cancer cases?
b. What signs or symptoms suggest cancer to you?
c. What actions do you take when you suspect cancer?
d. Are there any assessments or tests you use to confirm your suspicion?
e. If diagnosed as cancer, how do you proceed with treatment?

2. Have you ever advised a patient to seek care from an orthodox (hospital-based) provider for a breast problem?

Probes:

a. What was the reason behind your decision?
b. Did you follow up on the patient's condition afterward?

3. Is there anything else you would like to share regarding this topic or your experience?

Thank you very much for your participation.

Your contribution is greatly appreciated and will help improve care for women with breast cancer.

KWAME NKRUMAH UNIVERSITY OF SCIENCE & TECHNOLOGY

UNIVERSITY OF MINNESOTA
Driven to Discover®

TWI VERSION

Interview Guide

Me da w'ase sɛ w'agye ato mu sɛ wobɛhyia me. Me pɛ sɛ Manisa wo nsɛm bi a ayarefoɔ a wɔwɔ nufoɔ mu yareɛ a wohwɛ omo sɛ wɔyɛ dunsini yi. Wo nsɛmfua bɛboa yɛn na ama yɛn ate nnoɔma bi ase. Deɛ wobɛka akyerɛ me biara yɛ me ne wo asɛm a obiara nte bi na yɛde bɛyɛ nhwehwɛmu yi nko ara. Yɛde dɔnhwere baako na ebi di dwuma yi, yɛde begu afidie so na akyire no y'adane kasa no. Nsɛmmisa no gu ahodoɔ sedeɛ ɛbɛyɛ a yɛbɛtumi de ho nsɛm asie na yɛnhu wo pɔtenn. Ɛberɛ biara wotumi ma me gyae sɛ mede gu afidie so no. Megye ato mu. Yɛhyɛ nsɛmmisa no ase a mebisa wo nsɛm bebree, sedeɛ ɛbɛyɛ a mehu nnoɔma wofa mu sɛ wohwɛ omo a wɔwɔ nufoɔ mu yareɛ. Yɛni anoyie a ɛyɛ nokware anaa ɛnyɛ nokware. Deɛ wohunu no ha no sa na ɛteɛ. Mebisa asɛm na wompɛ sɛ wobɛyi ano a, megye ato mu. Wei kyerɛ sɛ ɛnyi asɛm ano sɛ wompɛ a. Wobɛtumi agyae nsɛm no anoyie berɛ biara.

Adunsifoɔ nsɛmmisa

Frɛ ano yi ni no wɔ ____________________ Da **ko** ________
Mfeɛ ________________

Sɛ woyɛ obaa /barima _____________

Kuro a wote ______________

Wo som _______________

Sɛ wowɔ ohokani anaa woni bi ______________

Wo mma dodoɔ _______________

W'akɔ sukuu aduru sɛn -------------------------------------

Mfeɛ sɛn ni_______________

Bea a woyɛ adwuma ____________

Adwumakuo a wowɔ mu wɔ adwuma hɔ ________________

KWAME NKRUMAH UNIVERSITY OF SCIENCE & TECHNOLOGY | UNIVERSITY OF MINNESOTA Driven to Discover®

Mfeɛ dodoɔ a w'ayɛ adwuma ________________

Wo di berɛ ____________________

Wowɔ nteteɛ soronko bi ---

Aane a, mepa wo kyɛw ka ne mfeɛ dodoɔ a wode wieyɛ ------------------------------

Adunsifoɔ nsɛmmisa

16. W'adi mfeɛ sɛn ? (Mfeɛ dodoɔ)……………………………………………………
17. Mfeɛ sɛn ni na w'ayɛ dunsini adwuma yi? (mfeɛ) ………………………
18. Ɛsom bɛn na wo som?
    d. Kristoni [ ]
    e. Kramoni [ ]
    f. Amammerɛ mu som [ ]
19. W'akɔ sukuu aduru sɛn?
    e. W'ankɔ sukuu [ ]
    f. "Primary education" [ ]
    g. "Secondary education" [ ]
    h. "Tertiary education"[ ]
20. Wowɔ nimdeɛ foforɔ ka nhaban dwuma yi ho?
    c. Aane [ ]
    d. Daabi [ ]
21. Sɛ Aane a, kyerɛ yɛn?
    ………………………………………………………………………
22. Amammerɛ kwan bɛn so na wofa sa yareɛ?
    d. Nhaban [ ]
    e. Sunsum (e.g., akɔm, afɔreɛ/apaeɛ) [ ]
    f. Biribi foforɔ wɔ hɔ a ka ……………………………………..
23. Wowɔ nteteɛ wɔ nhaban/ sunsum nnuro ho?
    c. Aane [ ]
    d. Daabi [ ]
24. Sɛ aane a, nteteɛ bɛn na wowɔ?
    e. Fri me busua ni hɔ (e.g., agya, nanabarima, etc) [ ]
    f. Nam daeɛ/ yi kyerɛ mu [ ]
    g. Sua no sukuu [ ]
    h. Biribi foforɔ a ka no ha……………………………………………
25. W'akɔ nteteɛ dwumadie bi a y'ayɛ ama wɔn a wɔde nhaban sa yadeɛ da?
    c. Aane[ ]
    d. Daabi[ ]
26. Wowɔ yareɛ pɔten a wode nhaban no sa?
    a. Aane [ ]
    b. Daabi [ ]

27. Deɛn ade paa na woyɛ ho adwuma?
…………………………………………………………………………………………………
…………………………………………………………………………………………………
28. Mmaa dodoɔ sɛn na wohwɛ omo bosome mu?…………………………………
29. Ayaresafoɔ dodoɔ sɛn na wohwɛ omo bosome mu?
…………………………………………………………………………….
30. Wo twerɛ ayaresafoɔ dodoɔ a wohwɛ wɔn?
    c. Aane [ ]
    d. Daabi [ ]
31. Sika sɛn na wogye sɛ wohwɛ ɔyarefoɔ a?
……………………………………………………………………………..
32. Woyi sika fri hɔ a, wogye biribi foforɔ bi?
    c. Aane [ ]
    d. Daabi [ ]
33. Sɛ aane a, deɛn na wogye sɛ w'antua sika a?
…………………………………………………………………………………………………
…………………………………………………………………………………………………
…………………………………………………………………………………………………
34. W'ama obi a wɔwɔ kokoram akɔ ayaresabea da??
    c. Aane[ ]
    d. Daabi [ ]
35. Aane a, bosome biara dodoɔ sɛn na woma wɔn kɔ?
……………………………………………

Nsɛmmisa

Asɛm titire

Sɛ ɔbaa de nufoɔ ho asɛm ba a, deɛn na wode yɛ nhwehwɛmu sɛ ɔwɔ yaadeɛ wei anaa sɛ wei ?

<u>Nhwehwɛmu</u>

1. Woyɛ deɛn hunu sɛ ɛyɛ kokoram anaa ɛnyɛ kokoram?
2. Deɛn na wohwɛ?
3. Deɛn na woyɛ sɛ wosusu sɛ ɛyɛ kokoram a?
    - Wo yɛ nhwehwɛmu bi?
    - Sɛ ɛyɛ kokoram a, anammɔn bɛn na wo tu?
4. Obi aba wo hɔ ayaresa ma wode ama dunsini foforɔ da?

- kyerɛkyerɛ wo gyinaeɛ mu
- Wodii akyire hwɛ sedeɛ ayaresa no si ɛkɔ?

5. Ɛbɛyɛ deɛn na adunsifoɔ ne wɔn a wɔyɛ sunsum adwuma aka abom asa nufoɔ mu kokoram yareɛ?

6. Wowɔ ɔhaw bi sɛ ɛba sɛ mobɛyɛ adwuma abɔ mu a?

7. Ɔkwan bɛn so na adunsifoɔ anaa wɔn a wɔsa sunsum yareɛ de hwɛ nufoɔ mu kokoram?

8. Wowɔ biribi foforɔ bi ka?

Medaase paa sɛ wode wo ho hyɛɛ dwumadie yi mu!
