# Supplementary material for: Gatekeepers or barriers? Exploring traditional medicine practitioners’ understanding of breast cancer in Ghana
Source: PLoS One. 2026 Aug 5;21(8):e0355289. doi: 10.1371/journal.pone.0355289 (PMC13440859; doi:10.1371/journal.pone.0355289)
Supplement: Supplementary file 2 [file pone.0355289.s002.pdf]

Kwame Nkrumah
University of Science
and Technology, Kumasi

College of Health Sciences

DEPARTMENT OF NURSING

Editor
PLOS One

26th June 2026

Dear Editor,

<u>Response to Reviewers – Revised Manuscript Submission</u>

Manuscript Title: Gatekeepers or barriers? Exploring traditional medicine practitioners' understanding of breast cancer in Ghana

Submission ID: PONE-D-26-03860

Thank you for the opportunity to revise and resubmit our manuscript. We are grateful to you and the reviewers for the insightful and constructive feedback provided. We have carefully considered all comments and have revised the manuscript substantially in response. We have prepared a detailed, point-by-point response outlining how each reviewer's comment has been addressed.

We appreciate your time and consideration of our revised manuscript.

Sincerely,

Adwoa Bemah Boamah Mensah, PhD (Corresponding Author)
Department of Nursing, Kwame Nkrumah University of Science and Technology
Email: bbemahc2000@gmail.com
On behalf of all co-authors

SN #	Section/Area	Comment	Authors' Response
Journal requirements:	PLOS ONE's style requirements	1. Please ensure that your manuscript meets PLOS ONE's style requirements, including those for file naming.	Thank you for this directive. We have formatted the manuscript according to PLOS ONE's style requirements, including those for file naming.
Reviewer # 1	Analysis	1. This is a great paper focusing on an under-reported topic.<br><br>There are a few places in the manuscript that refer to themes 'emerging' (method - line 122, line 151; results - line 208, 238). Please rephrase as themes do not emerge (see https://journals.sagepub.com/doi/10.1177/14733250241277355).	Thank you for this helpful comment and for directing us to the recent editorial by Morriss on the use of Braun and Clarke's thematic analysis. We agree that our previous wording could have implied that themes passively "emerged" from the data rather than being actively developed through researcher engagement and interpretation. We have therefore revised the manuscript to avoid this language throughout. Specifically, we replaced phrases such as "themes emerged" and "themes to emerge inductively" with wording such as "our analysis generated," "we developed," and "themes were developed through iterative engagement with the transcripts." We also revised the sampling and data analysis sections to avoid the phrase "no new themes emerged," instead describing the adequacy and depth of the dataset. Please refer to "Abstract" line 42 and "Data Analysis", lines 145 to 161.
	Specific comments	2. Please also avoid quantitative language (e.g.	Thank you for this important observation. We have reviewed the manuscript and revised language that

		'validated' on line 155).	could be read as overly quantitative, including the term "validated." For example, we replaced "interpretations were validated through team debriefings" with "interpretations were strengthened through team debriefings," and revised "participant validation" to "member checking." We also replaced "consensus was reached" with language describing discussion of differing interpretations and refinement of the analytic account. Please see "Trustworthiness", lines 162 to171 of the revised manuscript.
		3. In the results I was hoping for further information on what happens after recognition/suspicion of cancer or if/how beliefs about aetiology affect management. Indeed the discussed notes that beliefs might delay timely diagnosis. But at present there is no evidence of this presented in the results. Was there any data on this? If not available this should be recognised as a limitation.	Thank you for this helpful comment. We agree that the previous Results section did not sufficiently describe what happened after TMPs recognized or suspected breast cancer, nor how beliefs about aetiology may shape management. We have revised Theme #4 in the Results section to include an additional analytic paragraph on "*management responses after recognition or suspicion*". This paragraph shows that some TMPs' spiritual or social explanations were linked to advice about spiritual or relational repair, while bodily explanations were linked to treating perceived underlying causes such as reproductive or infectious problems. We have also included examples showing that some TMPs relied on biomedical confirmation through scans or hospital reports. Please refer to lines 336 to 356 of the revised manuscript.<br><br>However, we also recognize that the interviews did not systematically trace full patient pathways after TMP consultation. We have therefore added this as a limitation (lines 448 to 455)

			and revised the Discussion to avoid implying direct evidence that TMP beliefs caused diagnostic delay. Instead, we now present this as a possible mechanism that requires further study. Please refer to lines 380 to 391 of the revised manuscript.
