# Supplementary material for: Gatekeepers or barriers? Exploring traditional medicine practitioners’ understanding of breast cancer in Ghana
Source: PLoS One. 2026 Aug 5;21(8):e0355289. doi: 10.1371/journal.pone.0355289 (PMC13440859; doi:10.1371/journal.pone.0355289)
Supplement: Peer review history

# Peer review: Decision Letter 0

**DOI:** 10.1371/journal.pone.0355289.r001

**Authors:** Miquel Vall-llosera Camps

23 Jun 2026

PONE-D-26-03860

Gatekeepers or barriers? Exploring traditional medicine practitioners’ understanding of breast cancer in Ghana

PLOS One

Dear Dr. BOAMAH MENSAH,

Thank you for submitting your manuscript to PLOS ONE. After careful consideration, we feel that it has merit but does not fully meet PLOS ONE’s publication criteria as it currently stands. Therefore, we invite you to submit a revised version of the manuscript that addresses the points raised during the review process.

Please submit your revised manuscript by Aug 06 2026 11:59PM. If you will need more time than this to complete your revisions, please reply to this message or contact the journal office at plosone@plos.org. When you’re ready to submit your revision, log on to https://www.editorialmanager.com/pone/ and select the 'Submissions Needing Revision' folder to locate your manuscript file.

Please include the following items when submitting your revised manuscript:

- A letter that responds to each point raised by the academic editor and reviewer(s). You should upload this letter as a separate file labeled 'Response to Reviewers'.
- A marked-up copy of your manuscript that highlights changes made to the original version. You should upload this as a separate file labeled 'Revised Manuscript with Track Changes'.
- An unmarked version of your revised paper without tracked changes. You should upload this as a separate file labeled 'Manuscript'.

If you would like to make changes to your financial disclosure, please include your updated statement in your cover letter. Guidelines for resubmitting your figure files are available below the reviewer comments at the end of this letter.

If applicable, we recommend that you deposit your laboratory protocols in protocols.io to enhance the reproducibility of your results. Protocols.io assigns your protocol its own identifier (DOI) so that it can be cited independently in the future. For instructions see: https://journals.plos.org/plosone/s/submission-guidelines#loc-laboratory-protocols. Additionally, PLOS ONE offers an option for publishing peer-reviewed Lab Protocol articles, which describe protocols hosted on protocols.io. Read more information on sharing protocols at https://plos.org/protocols?utm_medium=editorial-email&utm_source=authorletters&utm_campaign=protocols.

As the corresponding author, your ORCID iD is verified in the submission system and will appear in the published article. PLOS supports the use of ORCID, and we encourage all coauthors to register for an ORCID iD and use it as well. Please encourage your coauthors to verify their ORCID iD within the submission system before final acceptance, as unverified ORCID iDs will not appear in the published article. *Only*  the individual author can complete the verification step; PLOS staff *cannot*  verify ORCID iDs on behalf of authors.

We look forward to receiving your revised manuscript.

Kind regards,

Miquel Vall-llosera Camps

Senior Staff Editor

PLOS One

Journal requirements:

When submitting your revision, we need you to address these additional requirements.

1. Please ensure that your manuscript meets PLOS ONE's style requirements, including those for file naming. The PLOS ONE style templates can be found at

https://journals.plos.org/plosone/s/file?id=wjVg/PLOSOne_formatting_sample_main_body.pdf and

https://journals.plos.org/plosone/s/file?id=ba62/PLOSOne_formatting_sample_title_authors_affiliations.pdf

If the reviewer comments include a recommendation to cite specific previously published works, please review and evaluate these publications to determine whether they are relevant and should be cited. There is no requirement to cite these works unless the editor has indicated otherwise.

Please review your reference list to ensure that it is complete and correct. If you have cited papers that have been retracted, please include the rationale for doing so in the manuscript text, or remove these references and replace them with relevant current references. Any changes to the reference list should be mentioned in the rebuttal letter that accompanies your revised manuscript. If you need to cite a retracted article, indicate the article’s retracted status in the References list and also include a citation and full reference for the retraction notice.

[Note: HTML markup is below. Please do not edit.]

Reviewers' comments:

Reviewer's Responses to Questions

**Comments to the Author**

1. Is the manuscript technically sound, and do the data support the conclusions?

The manuscript must describe a technically sound piece of scientific research with data that supports the conclusions. Experiments must have been conducted rigorously, with appropriate controls, replication, and sample sizes. The conclusions must be drawn appropriately based on the data presented.

Reviewer #1: Yes

**********

2. Has the statistical analysis been performed appropriately and rigorously?

Reviewer #1: N/A

**********

3. Have the authors made all data underlying the findings in their manuscript fully available?

The PLOS Data policy requires authors to make all data underlying the findings described in their manuscript fully available without restriction, with rare exception (please refer to the Data Availability Statement in the manuscript PDF file). The data should be provided as part of the manuscript or its supporting information, or deposited to a public repository. For example, in addition to summary statistics, the data points behind means, medians and variance measures should be available. If there are restrictions on publicly sharing data—e.g. participant privacy or use of data from a third party—those must be specified.

Reviewer #1: Yes

**********

4. Is the manuscript presented in an intelligible fashion and written in standard English?

PLOS ONE does not copyedit accepted manuscripts, so the language in submitted articles must be clear, correct, and unambiguous. Any typographical or grammatical errors should be corrected at revision, so please note any specific errors here.

Reviewer #1: Yes

**********

5. Review Comments to the Author

Please use the space provided to explain your answers to the questions above. You may also include additional comments for the author, including concerns about dual publication, research ethics, or publication ethics. (Please upload your review as an attachment if it exceeds 20,000 characters)

Reviewer #1: This is a great paper focusing on an under-reported topic.

There are a few places in the manuscript that refer to themes 'emerging' (method - line 122, line 151; results - line 208, 238). Please rephrase as themes do not emerge (see https://journals.sagepub.com/doi/10.1177/14733250241277355 ).

Please also avoid quantitative language (e.g. 'validated' on line 155).

In the results I was hoping for further information on what happens after recognition/suspicion of cancer or if/how beliefs about aetiology affect management. Indeed the discussed notes that beliefs might delay timely diagnosis. But at present there is no evidence of this presented in the results. Was there any data on this? If not available this should be recognised as a limitation.

**********

6. PLOS authors have the option to publish the peer review history of their article (what does this mean?). If published, this will include your full peer review and any attached files.

If you choose “no”, your identity will remain anonymous but your review may still be made public.

**Do you want your identity to be public for this peer review?**  For information about this choice, including consent withdrawal, please see our Privacy Policy.

Reviewer #1: **Yes:**  Suzanne Scott

**********

[NOTE: If reviewer comments were submitted as an attachment file, they will be attached to this email and accessible via the submission site. Please log into your account, locate the manuscript record, and check for the action link "View Attachments". If this link does not appear, there are no attachment files.]

To ensure your figures meet our technical requirements, please review our figure guidelines: https://journals.plos.org/plosone/s/figures

You may also use PLOS’s free figure tool, NAAS, to help you prepare publication quality figures: https://journals.plos.org/plosone/s/figures#loc-tools-for-figure-preparation.

NAAS will assess whether your figures meet our technical requirements by comparing each figure against our figure specifications.

# Author comment: Author response to Decision Letter 1

**DOI:** 10.1371/journal.pone.0355289.r002

26 Jun 2026

Journal requirements: 1. Please ensure that your manuscript meets PLOS ONE's style requirements, including those for file naming.

Author's Response: Thank you for this directive. We have formatted the manuscript according to PLOS ONE's style requirements, including those for file naming.

Reviewer # 1

1. 1. This is a great paper focusing on an under-reported topic.

There are a few places in the manuscript that refer to themes 'emerging' (method - line 122, line 151; results - line 208, 238). Please rephrase, as themes do not emerge (see https://journals.sagepub.com/doi/10.1177/14733250241277355 ).

Author's Response: Thank you for this helpful comment and for directing us to the recent editorial by Morriss on the use of Braun and Clarke’s thematic analysis. We agree that our previous wording could have implied that themes passively “emerged” from the data rather than being actively developed through researcher engagement and interpretation. We have therefore revised the manuscript to avoid this language throughout. Specifically, we replaced phrases such as “themes emerged” and “themes to emerge inductively” with wording such as “our analysis generated,” “we developed,” and “themes were developed through iterative engagement with the transcripts.” We also revised the sampling and data analysis sections to avoid the phrase “no new themes emerged” and instead describe the dataset's adequacy and depth. Please refer to "Abstract," line 42, and “Data Analysis," lines 145 to 161.

2. Please also avoid quantitative language (e.g., 'validated' on line 155).

Author's Response: Thank you for this important observation. We have reviewed the manuscript and revised language that could be read as overly quantitative, including the term “validated.” For example, we replaced “interpretations were validated through team debriefings” with “interpretations were strengthened through team debriefings" and revised “participant validation” to “member checking.” We also replaced “consensus was reached” with language describing discussion of differing interpretations and refinement of the analytic account. Please see "Trustworthiness," lines 162 to 171 of the revised manuscript.

3. In the results I was hoping for further information on what happens after recognition/suspicion of cancer or if/how beliefs about aetiology affect management. Indeed, the notes discuss that beliefs might delay timely diagnosis. But at present there is no evidence of this presented in the results. Was there any data on this? If not available, this should be recognized as a limitation.

Author's Response: Thank you for this helpful comment. We agree that the previous Results section did not sufficiently describe what happened after TMPs recognized or suspected breast cancer, nor how beliefs about etiology may shape management. We have revised Theme #4 in the Results section to include an additional analytic paragraph on “management responses after recognition or suspicion." This paragraph shows that some TMPs’ spiritual or social explanations were linked to advice about spiritual or relational repair, while bodily explanations were linked to treating perceived underlying causes such as reproductive or infectious problems. We have also included examples showing that some TMPs relied on biomedical confirmation through scans or hospital reports. Please refer to lines 336 to 356 of the revised manuscript.

However, we also recognize that the interviews did not systematically trace full patient pathways after TMP consultation. We have, therefore, added this as a limitation (lines 448 to 455) and revised the Discussion to avoid implying direct evidence that TMP beliefs caused diagnostic delay. Instead, we now present this as a possible mechanism that requires further study. Please refer to lines 380 to 391 of the revised manuscript.

Attachment Submitted filename: RESPONSE TO REVIWERS.pdf

# Peer review: Decision Letter 1

**DOI:** 10.1371/journal.pone.0355289.r003

**Authors:** Sawsan Al-Rawi

20 Jul 2026

Gatekeepers or barriers? Exploring traditional medicine practitioners’ understanding of breast cancer in Ghana

PONE-D-26-03860R1

Dear Dr. BOAMAH MENSAH,

We’re pleased to inform you that your manuscript has been judged scientifically suitable for publication and will be formally accepted for publication once it meets all outstanding technical requirements.

Within one week, you’ll receive an e-mail detailing the required amendments. When these have been addressed, you’ll receive a formal acceptance letter and your manuscript will be scheduled for publication.

An invoice will be generated when your article is formally accepted. Please note, if your institution has a publishing partnership with PLOS and your article meets the relevant criteria, all or part of your publication costs will be covered. Please make sure your user information is up-to-date by logging into Editorial Manager at Editorial Manager® and clicking the ‘Update My Information' link at the top of the page. For questions related to billing, please contact billing support.

If your institution or institutions have a press office, please notify them about your upcoming paper to help maximize its impact. If they’ll be preparing press materials, please inform our press team as soon as possible -- no later than 48 hours after receiving the formal acceptance. Your manuscript will remain under strict press embargo until 2 pm Eastern Time on the date of publication. For more information, please contact onepress@plos.org.

Kind regards,

Sawsan S Al-Rawi

Guest Editor

PLOS One

Additional Editor Comments (optional):

Reviewers' comments:

Reviewer's Responses to Questions

**Comments to the Author**

1. If the authors have adequately addressed your comments raised in a previous round of review and you feel that this manuscript is now acceptable for publication, you may indicate that here to bypass the “Comments to the Author” section, enter your conflict of interest statement in the “Confidential to Editor” section, and submit your "Accept" recommendation.

Reviewer #1: All comments have been addressed

**********

2. Is the manuscript technically sound, and do the data support the conclusions?

The manuscript must describe a technically sound piece of scientific research with data that supports the conclusions. Experiments must have been conducted rigorously, with appropriate controls, replication, and sample sizes. The conclusions must be drawn appropriately based on the data presented.

Reviewer #1: Yes

**********

3. Has the statistical analysis been performed appropriately and rigorously?

Reviewer #1: N/A

**********

4. Have the authors made all data underlying the findings in their manuscript fully available?

The PLOS Data policy requires authors to make all data underlying the findings described in their manuscript fully available without restriction, with rare exception (please refer to the Data Availability Statement in the manuscript PDF file). The data should be provided as part of the manuscript or its supporting information, or deposited to a public repository. For example, in addition to summary statistics, the data points behind means, medians and variance measures should be available. If there are restrictions on publicly sharing data—e.g. participant privacy or use of data from a third party—those must be specified.

Reviewer #1: No

**********

5. Is the manuscript presented in an intelligible fashion and written in standard English?

PLOS ONE does not copyedit accepted manuscripts, so the language in submitted articles must be clear, correct, and unambiguous. Any typographical or grammatical errors should be corrected at revision, so please note any specific errors here.

Reviewer #1: Yes

**********

6. Review Comments to the Author

Please use the space provided to explain your answers to the questions above. You may also include additional comments for the author, including concerns about dual publication, research ethics, or publication ethics. (Please upload your review as an attachment if it exceeds 20,000 characters)

Reviewer #1: (No Response)

**********

7. PLOS authors have the option to publish the peer review history of their article (what does this mean?). If published, this will include your full peer review and any attached files.

If you choose “no”, your identity will remain anonymous but your review may still be made public.

**Do you want your identity to be public for this peer review?**  For information about this choice, including consent withdrawal, please see our Privacy Policy.

Reviewer #1: **Yes:**  Suzanne Scott

**********

# Editor report: Acceptance letter

**DOI:** 10.1371/journal.pone.0355289.r004

**Authors:** Sawsan Al-Rawi

PONE-D-26-03860R1

PLOS One

Dear Dr. Boamah Mensah,

I'm pleased to inform you that your manuscript has been deemed suitable for publication in PLOS One. Congratulations! Your manuscript is now being handed over to our production team.

At this stage, our production department will prepare your paper for publication. This includes ensuring the following:

* All references, tables, and figures are properly cited

* All relevant supporting information is included in the manuscript submission,

* There are no issues that prevent the paper from being properly typeset

You will receive further instructions from the production team, including instructions on how to review your proof when it is ready. Please keep in mind that we are working through a large volume of accepted articles, so please give us a few days to review your paper and let you know the next and final steps.

Lastly, if your institution or institutions have a press office, please let them know about your upcoming paper now to help maximize its impact. If they'll be preparing press materials, please inform our press team within the next 48 hours. Your manuscript will remain under strict press embargo until 2 pm Eastern Time on the date of publication. For more information, please contact onepress@plos.org.

You will receive an invoice from PLOS for your publication fee after your manuscript has reached the completed accept phase. If you receive an email requesting payment before acceptance or for any other service, this may be a phishing scheme. Learn how to identify phishing emails and protect your accounts at https://explore.plos.org/phishing.

If we can help with anything else, please email us at customercare@plos.org.

Thank you for submitting your work to PLOS ONE and supporting open access.

Kind regards,

PLOS ONE Editorial Office Staff

on behalf of

Mrs. Sawsan S Al-Rawi

Guest Editor

PLOS One
